# Relationship between maternal serotonin levels and autism-associated genetic variants

**DOI:** 10.1172/JCI179238

**Published:** 2024-07-04

**Authors:** Amandeep Jutla, Lauren C. Shuffrey, Stephen J. Guter, Kally C. O’Reilly, George M. Anderson, James S. Sutcliffe, Edwin H. Cook, Jeremy Veenstra-VanderWeele

**Affiliations:** 1 Columbia University, New York, New York, USA.; 2 New York State Psychiatric Institute, New York, New York, USA.; 3 New York University, New York, New York, USA.; 4 University of Illinois Chicago (UIC), Chicago, Illinois, USA.; 5 Child Study Center, Yale University School of Medicine, New Haven, Connecticut, USA.; 6 Vanderbilt University, Nashville, Tennessee, USA.

**Keywords:** Neuroscience, Genetic variation

**To the Editor:** The serotonin system is implicated in neurodevelopment. For example, maternal disruption of serotonin genes in rodents produces offspring neurodevelopmental changes (1). In humans, lower maternal whole blood 5-hydroxytryptamine (WB5-HT) associates with greater neurodevelopmental severity in autistic children (2). Family and twin data support the idea that multiple common genetic and nongenetic factors together contribute to autism. However, a subgroup (10%–20%) carry a single rare, de novo genetic variant that contributes the vast majority of their autism risk (3). These rare autism-associated variants are most often found in autistic individuals with more severe neurodevelopmental phenotypes. Whether rare autism-associated genetic variants are associated with maternal serotonin levels has not previously been investigated. We therefore compared maternal WB5-HT levels in mothers of probands with rare autism-associated genetic variants to mothers whose autistic children did not harbor known rare genetic variants (Figure 1A).

Our sample comprised 276 children with maternal WB5-HT data, 35 (13%) of whom had at least one rare autism-associated genetic variant (Supplemental Table 1). Inheritance was de novo in 32 (91%), maternal in two (6%), and paternal in one (3%). Sample demographics (Supplemental Table 2) were consistent with those typically reported in autism samples: most were male (n = 229, 86%), White (n = 205, 78%), and non-Latine (n = 231, 88%).

We did not find a significant difference in median maternal WB5-HT between autistic children with and without a rare variant (W = 4897.5, P = 0.12); however, we did find that maternal WB5-HT was lower in rare variant carriers at the 75th (by 33.07, 95% CI = 5.55–64.40, P = 0.02) and 90th (by 64.60, 95% CI 26.65–101.44, P < 0.01) percentiles (Figure 1B and Supplemental Table 3). Similar analyses in ancillary samples of 301 children with paternal WB5-HT data (n = 301, 34 with a rare variant) and 346 with child WB5-HT data (n = 346, 38 with a rare variant) did not find median or quantile-level differences in WB5-HT between rare variant carriers and noncarriers (Figure 1, C and D).

Even in this modest sample size, we observed that WB5-HT extended into a higher range in mothers of autistic children without rare genetic variants as compared with mothers of children with variants. Although this aligns with our hypothesis of relatively lower WB5-HT in mothers of probands with rare variants, we did not see significant differences at a group median level. We observed no differences in fathers or autistic individuals themselves, indicating that these effects are not simply due to the heritability of WB5-HT.

The skewness of the maternal WB5-HT distribution in noncarriers indicates that the highest quantiles, where differences were observed, deviated from an expected normal distribution. This skewness in maternal WB5-HT parallels the elevated WB5-HT consistently observed in approximately 25% of autistic individuals. This may suggest that elevated maternal WB5-HT is associated with autism risk in a subset of individuals without rare genetic variants. We lacked control families that would have allowed us to evaluate maternal WB5-HT in comparison with general population WB5-HT (Figure 1A).

Additional limitations include modest statistical power due to the small number of probands carrying a genetic variant. Further, maternal WB5-HT was assessed after autism diagnosis, rather than during pregnancy, which would presumably be the time point when maternal WB5-HT would have an impact on offspring neurodevelopment. Our sample’s inclusion of only probands with an autism diagnosis also prevented the evaluation of a general impact of maternal WB5-HT on offspring development. Future work should include prospective studies of maternal WB5-HT during pregnancy with longitudinal follow-up in offspring, including in relation to autistic and other neurodevelopmental traits.

Samples of autistic probands and their parents are from the UIC Autism Center of Excellence (ACE) (4) and the UIC and Vanderbilt University sites of the Simons Simplex Collection (SSC) (5). Neither ACE nor SSC samples included nonautistic control probands or their parents.

WB5-HT was assayed using HPLC (2). Individuals taking medications known to affect 5-HT were excluded. Exome sequencing identified individuals with rare genetic variants.

After identifying a difference in skewness (Supplemental Figure 1), we compared median maternal WB5-HT via a two-sample Wilcoxon’s rank-sum test. We next compared maternal WB5-HT across groups at five quantiles (the 10th, 25th, 50th, 75th, and 90th percentiles) using a percentile bootstrap method that accounts for multiple comparisons in conjunction with the Harrell-Davis quantile comparison estimator (6).

This study was approved by the Institutional Review Boards of UIC and Vanderbilt University. Informed consent was obtained from all participants.

Values for all data points in graphs are reported in the Supporting Data Values file. Code is available at https://github.com/amandeepjutla/2023-5ht-variants/commit/3a12c182e8d78aa75527ebbdc80120f32eea2344 SSC and ACE data are, respectively, available from the Simons Foundation Autism Research Initiative (SSC Phenotype Dataset Version 15) and the National Institute of Mental Health (NIMH) Data Archive (https://nda.nih.gov/study.html?id=416).

## Supplementary Material

Supplemental data

Supporting data values

## Figures and Tables

**Figure 1:**
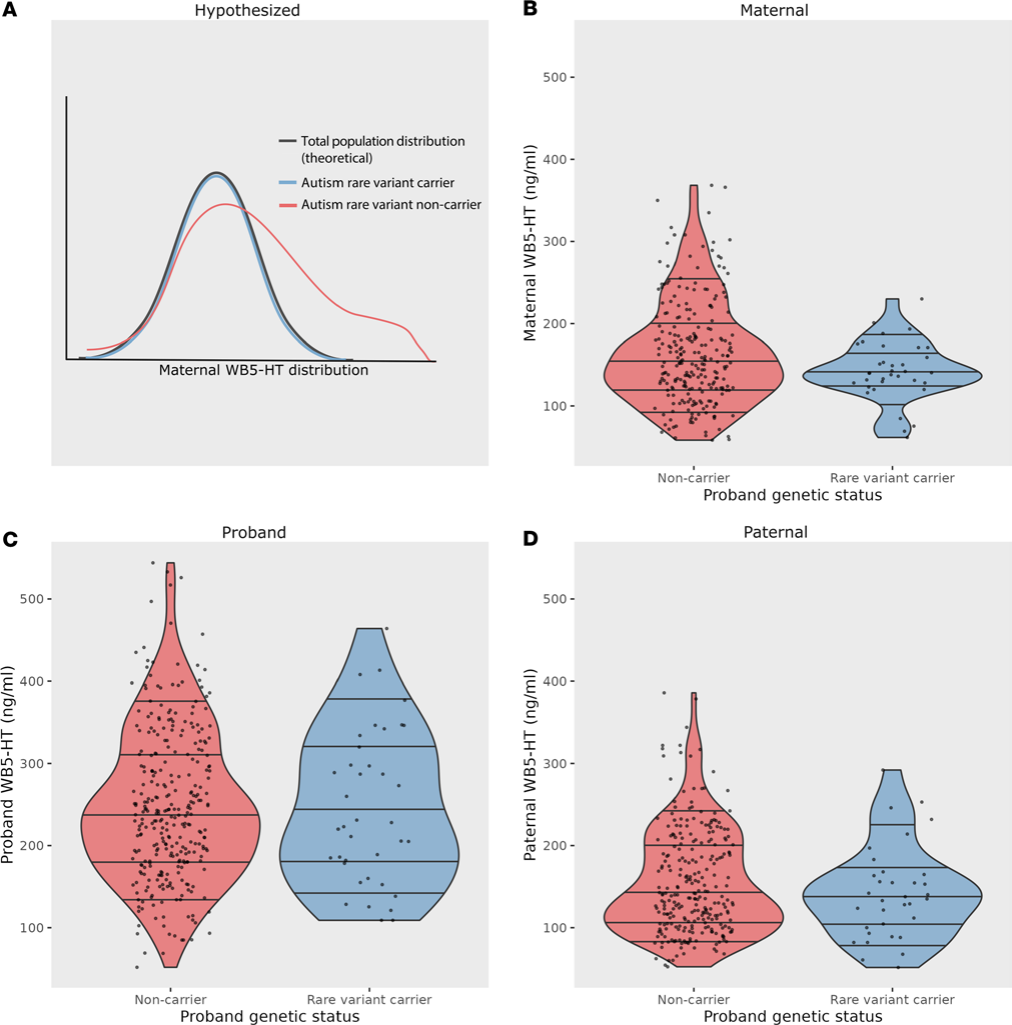
Comparative maternal, proband, and paternal WB5-HT levels between autism rare variant carriers and noncarriers. We predicted that the mothers of probands carrying rare autism-associated variants would have lower WB5-HT than mothers of noncarrier probands. With the observation that 5-HT levels in mothers of ASD probands show an upward skew (2), we hypothesized that the mothers of noncarrier probands would not show this skew and would instead show a normal distribution that would largely overlap with the theoretical distribution of the general population (**A**). Although median maternal WB5-HT did not differ across groups, the distribution was significantly upward shifted in the autism rare-variant noncarriers (**B**). We observed no significant differences in proband (**C**) or paternal (**D**) WB5-HT.

## Version 1 07/04/2024

In-Press Preview

## Version 2 09/03/2024

Electronic publication
