# Supplementary material for: Relationship between maternal serotonin levels and autism-associated genetic variants
Source: J Clin Invest. 2024 Jul 4;134(17):e179238. doi: 10.1172/JCI179238 (PMC11364378; doi:10.1172/JCI179238)
Supplement: Supplemental data [file jci-134-179238-s033.pdf]

Supplemental Figure 1

We calculated the skewness of the maternal WB5-HT distribution in each group (n = 35 probands with a rare variant and n = 276 without) using the standard formula $\frac{n}{(n-1)(n-2)}\sum\left(\frac{x_i - \bar{x}}{s}\right)^3$. Non-carriers showed a pronounced right skew (0.80) and rare variant carriers showed a slight left skew (-0.10).

To assess whether skew in non-carriers was significantly greater, we used a resampling method to generate 10,000 bootstrap samples for each group, calculating skewness for each sample. We then calculated the 95% confidence interval (CI) of the skewness distribution for each group (rare variant carriers, 95% CI -0.82 – 0.70; non-carriers, 95% CI 0.53 – 1.05). Although these CIs overlap, the lower bound of the CI for rare variant carriers was smaller than the upper bound of the CI for non-carriers, supporting our directional hypothesis that maternal WB5-HT skewness was greater among non-carriers than carriers.

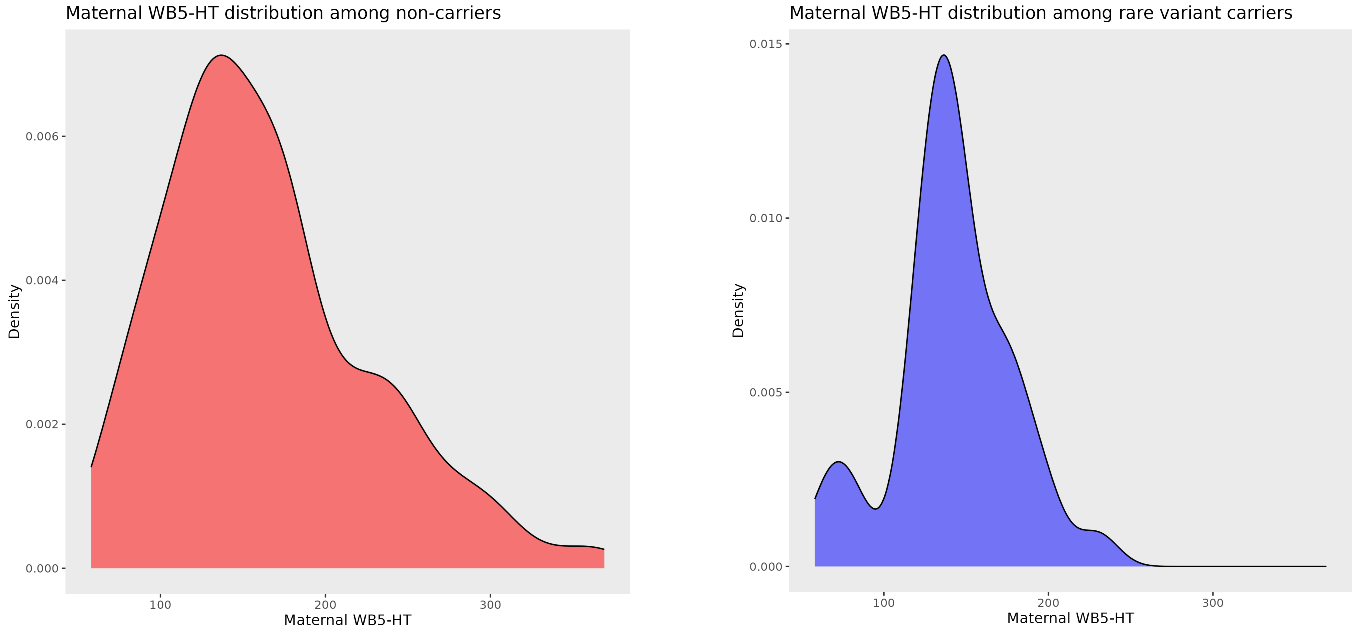

Supplemental Table 1

Genes and CNVs included in the rare variant group.

Gene	Inheritance*
ABCA2	
ABCA8	
ADCY5	
AGO2	
AMIGO1	
ANK2	
ARHGEF16	
BRAT1	
BRD4	
C9orf57	
CACNA1B	
CCDC37	
CD82	
CELF1	
CHAT	
CHD8	
CLCNKB	
COG3	
CPD	
CS	
DHX58	
DLX6	
DYRK1A	
ECEL1	
ELTD1	
EPRS	
FOXN1	
GFRA1	
GLP1R	
GNA11	
GRIK2	
HDAC1	
IL17RA	
ITGA5	
ITPR3	
KDM5B	
KIAA0100	
KIAA1244	
KLHL26	
KMT2B	
KMT2C	
LMTK3	
MAP3K13	
MUC22	
MYH7B	
NF1	
NR2F1	
NUP214	
PAPLN	
PASK	
PLEC	
PLEKHG2	
POLQ	
PPP1R9B	
PQBP1	Maternal
PRKAA1	
RANBP3	
RARA	
REN	
RIMBP3	
RSPH6A	
SCN2A	
SCN2A	
SPEN	
SPG7	
SPTBN1	
SRSF7	
SYNGAP1	
TM4SF18	
TMEM214	
TNPO3	
TTC16	
UGT2B10	
VSX2	
VWF	
WIPF1	
ZEB2	
ZFYVE26	
ZNF772	

CNV	Type	Inheritance*
chr1:93321461-113327865	Deletion	
chr15:21168391-26113882	Duplication	Maternal
chr15:21235224-26144736	Duplication	
chr16:15387380-18085470	Duplication	
chr16:29470951-30059319	Deletion	
chr16:29531748-30107306	Deletion	
chr16:29664595-30188229	Deletion	
chr16:6296188-6309514	Deletion	Paternal
chr16:8828382-9147487	Duplication	
chr17:6058839-7267958	Duplication	
chr2:103264154-108718935	Deletion	
chr2:61390488-61873147	Duplication	
chr3:197219312-198851029	Deletion	
chr3:67223272-72356021	Deletion	
chr3:67495701-67609694	Deletion	
chr7:73331560-74719039	Duplication	
chrX:1-154900000	Duplication	

* All variants were de novo except where otherwise indicated.

Supplemental Table 2

	Demographic characteristics of study participants by rare variant carrier status			
**Characteristic**	**Overall**, N = 276[1]	**Non-carrier**, N = 241[1]	**Carrier**, N = 35[1]	**p-value**[2]
Age in years	9.42 (4.17)	9.43 (4.12)	9.38 (4.52)	0.69
Study site				0.34
UIC	161 (58%)	138 (57%)	23 (66%)	
Vanderbilt	115 (42%)	103 (43%)	12 (34%)	
Sex				>0.99
Male	229 (86%)	199 (86%)	30 (88%)	
Female	36 (14%)	32 (14%)	4 (12%)	
Race				0.23
Black	33 (13%)	32 (14%)	1 (2.9%)	
White	205 (78%)	176 (77%)	29 (85%)	
Asian	9 (3.4%)	8 (3.5%)	1 (2.9%)	
More than one race	16 (6.1%)	13 (5.7%)	3 (8.8%)	
Ethnicity				0.093
Latine	33 (12%)	32 (14%)	1 (2.9%)	
Non-Latine	231 (88%)	198 (86%)	33 (97%)	

[1] Mean (SD); n (%)
[2] Wilcoxon rank sum test; Pearson's Chi-squared test; Fisher's exact test

Sex as a Biological Variable: Our study's examination of both maternal and paternal WB5-HT is based on biological sex. Biological sex was not otherwise incorporated into our analyses, but, as shown in the table above, sex data were collected from all participants. Our study, consistent with the demographics of autism, included more male than female participants. The sample size of females in the rare variant carrier group was too small to support a sex-specific analysis.

Supplemental Table 3

Maternal WB5-HT by quantile for those probands with a rare variant vs. those without

QU	*N* (no variant)	*N* (variant)	Median (no variant)	Median (variant)	Difference	CI Lower	CI Upper	*Adj. p*
0.10	241	35	91.04	90.58	0.46	−31.70	23.63	0.956
0.25	241	35	120.51	125.50	−5.00	−19.76	20.65	0.534
0.50	241	35	153.24	140.21	13.03	−5.04	27.76	0.065
**0.75**	**241**	**35**	**197.51**	**164.44**	**33.07**	**5.55**	**64.40**	**0.002**
**0.90**	**241**	**35**	**253.23**	**188.62**	**64.60**	**26.65**	**101.44**	**0.000**
